# Identification and application of anti-inflammatory compounds screening system based on RAW264.7 cells stably expressing NF-κB-dependent SEAP reporter gene

**DOI:** 10.1186/s40360-016-0113-6

**Published:** 2017-01-18

**Authors:** Yue Li, Xiaomeng Wang, Juan Ren, Xi Lan, Jing Li, Jing Yi, Li Liu, Yan Han, Sanqi Zhang, Dongmin Li, Shemin Lu

**Affiliations:** 1 Department of Biochemistry and Molecular Biology, School of Basic Medical Sciences, Xi’an Jiaotong University Health Science Centre, Xi’an, Shaanxi 710061 People’s Republic of China; 2 Key Laboratory of Environment and Genes Related to Diseases (Xi’an Jiaotong University), Ministry of Education of China, Xi’an, Shaanxi 710061 People’s Republic of China; 3 Department of Medicinal Chemistry, School of Pharmacy, Xi’an Jiaotong University Health Science Centre, Xi’an, Shaanxi 710061 People’s Republic of China; 4 Department of Reproductive Medicine, The Fourth Hospital of Xi’an, Xi’an, Shaanxi, People’s Republic of China

**Keywords:** Reporter gene, Inflammation, Drug screening, Signal pathway

## Abstract

**Background:**

NF-κB is one of the key transcription factors in the inflammatory response, transactivates a series of pro-inflammatory genes and is therefore regarded as an important target for anti-inflammatory drug screening.

**Method:**

We recombined the reporter gene vector with inserting the “neo” transcript into the vector pNF-κB-SEAP, made the reporter gene vector stable in a eukaryotic cell line. The recombinant reporter gene vector we named pNF-κB-SEAP-Neo was transfected into RAW264.7. We selected the transfected RAW264.7 cell line with G418 for 15 days and then get RAW264.7 cells stably expressing NF-κB-dependent SEAP named as RAW264.7-pNF-κB-SEAP cells. We treated the RAW264.7-pNF-κB-SEAP cells with NF-κB agonists as LPS, PolyI:C and TNF-α, NF-κB inhibitor as PDTC and BAY117085, in different concentrations and time points and tested the expression of the SEAP, constructed the drug screening system on the base of the RAW264.7-pNF-κB-SEAP cell line. 130 chemicals were screened with the drug screening system we constructed and one of these chemicals numbered w10 was found could inhibit the NF-κB significantly. At last, we verified the inhibition of w10 to expression of genes promoted with NF-κB in HepG2 and Hela, and to migration of Hela.

**Result:**

In this study, we established a drug screening system based on RAW264.7 cells that stably expressed the NF-κB-dependent, SEAP reporter gene. To develop a standard method for drug screening using this reporter-gene cell line, the test approach of SEAP was optimized and basic conditions for drug screening were chosen. This included the initial cell number inoculated in a 96-well plate, the optimum agonist, inhibitor of NF-κB pathway and their concentrations during screening. Subsequently, 130 newly synthesized compounds were screened using the stable reporter-gene cell line. The anti-inflammatory effects of the candidate compounds obtained were further verified in 2 cancer cell lines. The results indicated that compound W10 (methyl 4-(4-(prop-2-yn-1-ylcarbamoyl) phenylcarbamoyl) benzoate) significantly inhibited SEAP production under the screening conditions. Further results confirmed that the precursor compound significantly inhibited the transcription of NF-κB target genes.

**Conclusion:**

In conclusion, RAW264.7 cells, stably expressing the NF-κB-dependent SEAP-reporter gene, may provide a new, feasible, and efficient cellular drug-screening system.

**Electronic supplementary material:**

The online version of this article (doi:10.1186/s40360-016-0113-6) contains supplementary material, which is available to authorized users.

## Background

Inflammation is a defence response to infection, injury, and/or stress. When acute inflammatory response is triggered, it lasts for a short period and is regulated by negative feedback signals. Dysregulation of this feedback mechanism results in chronic inflammation, which is believed to be a key pathophysiological mechanism in various health disorders. In metabolic syndrome, for example, inflammation seems to be instrumental in the pathogenesis of insulin resistance and hepatic steatosis [1, 2], In various cancers, the inflammatory milieu provides a favourable condition for malignant cells to proliferate and migrate [3].

The macrophage, which is ubiquitously-distributed, plays an important role in inflammation. Its functions include phagocytosis, antigen presentation and secretion of different types of cytokines and chemokines to regulate inflammation. In insulin resistance associated with metabolic syndrome, macrophages secrete proinflammatory cytokines such as tumour necrosis factor (TNF)-α and interleukin (IL)-1β [4] while in primary and metastatic tumours, they provide a proinflammatory microenvironment for cancer cell growth [5]. Based on their role in inflammation, macrophage activation has been recognized as a target for anti-inflammation therapy. One of the signal transduction pathways involved during macrophage activation is the nuclear factor-κB (NF-κB) pathway.

NF-κB is a nuclear transcription factor and contains 5 subunits: c-Rel, RelA (p65), RelB, NF-κB1 (p50 and p105), and NF-κB2 (p52 and p100) [6]. Inactive NF-κB forms a trimer consisting of RelA, p50, and inhibitor protein IκB in the cytoplasm. During canonical activation IκB is phosphorylated, separates from the heterodimer RelA/p50 and is degraded via ubiquitination [7]. At the same time, the phosphorylated RelA/p50 dimer translocates into the nucleus, and binds with cis-acting transcription elements in its target genes [8] to form a transcription complex. NF-κB is known to activate the transcription of more than 400 genes [9]. During acute inflammation such as in bacterial infection, activation of NF-κB up-regulates transcription of various cytokines and chemokines that promote the inflammatory response and antigen presentation; while during chronic inflammation, NF-κB triggers the transcription of more complicated genes that are involved in growth, transformation, and survival of cells [10]. Another difference between acute and chronic inflammation is that NF-κB is activated for short and long periods, respectively [11]. In both, metabolic syndrome and cancer, NF-κB is activated continuously to promote transcription of several proinflammatory cytokines [12]. NF-κB also up-regulates proliferation and migration of tumour cells [6] and plays a major role in the inflammatory microenvironment of the tumour [3]. Besides being a direct trigger and regulator of the inflammatory response, NF-κB is itself regulated through crosstalk with other intracellular signalling pathways [13]. Therefore, macrophages and NF-κB are both considered as important cellular and molecular screening targets for anti-inflammation and anti-cancer drugs [14].

In drug screening, the strategy strikingly depends on the target choice and the assay method [15]. Recently, molecules of the signalling pathway have become new target choices for drug screening. Compared with targeting cellular receptors, signal pathway molecules represent the activation or inhibition of a compound within the signal pathway more directly, thus requiring identification of the mechanism of action of the screened compound. Regarding assay methods, secreted alkaline phosphatase (SEAP) is extensively used in the detection of positive candidates [16]. The gene encoding SEAP has been used as a reporter gene in screening and its activity is represented by SEAP-expression yield. SEAP catalyses p-nitrophenyl phosphate (PNPP) into 4-NPP, transforming to yellow and soluble quinones with maximum absorption wavelength at 405 nm [17]. Thus, SEAP activity can be measured in terms of concentration of quinones produced in presence of SEAP. SEAP has several advantages as a reporter gene for drug screening: feasibility of application within the culture supernatant, high stability at 65 °C, and prolonged half-life [18].

In this study, we constructed a drug screening system targeting the NF-κB pathway in macrophages. We produced a recombinant pNF-κB-SEAP vector, transfected mouse macrophages, and obtained a cell line stably expressing the reporter gene. We optimized conditions for the SEAP test and basic conditions of recombined cell culture and treatment with well-known activators and inhibitors of the NK-κB pathway. A total of 130 newly-synthesized compounds were screened using this drug screening system and 8 compounds displayed striking inhibitory activity for SEAP. Compound W10 was selected to evaluate the screening results in HeLa and HepG 2 cells, and the results showed that W10 significantly inhibited the expression of NF-κB targeting genes.

## Methods

### Recombination of vector pNF-κB-SEAP

**Fig. 1:**
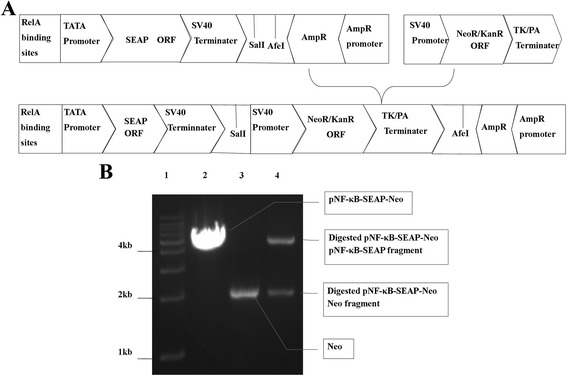
Recombined reporter gene vector pNF-κB-SEAP-Neo. **a** Schematic diagram of the recombined reporter gene vector pNF-κB-SEAP-Neo. The ORF of eukaryotic/ prokaryotic resistance gene NeoR/KanR was amplified from pEGFP-N1 vector and inserted into pNF-κB-SEAP vector between 2 restriction sites of SalI and AfeI to construct the recombined vector pNF-κB-SEAP-Neo. **b** Identification of recombined reporter gene vector pNF-κB-SEAP-Neo. Double-restriction-enzyme digestion analysis of SalI and AfeI was performed with 1% agarose gel electrophoresis. Lane 1 is the 1-kb ladder marker. Lane 2 is the restriction enzyme-digested products of the reporter gene vector pNF-κB-SEAP. Lane 3 is the restriction enzyme-digested products of amplified “Neo” transcript. Lane 4 is the restriction enzyme-digested products of the recombined reporter gene pNF-κB-SEAP-Neo

To recombine the vector pNF-κB-SEAP to be stable in eukaryocyte, we analysed the vector sequence (http://www.addgene.org) and found that there are 2 restriction sites (SalI and AfeI) downstream of the SEAP transcript. The vector pEGFP-N1 (Clontech) includes the sequence of neomycin resistance gene “neo”. Therefore, the open reading frame for “neo” was first amplified (forward primer: 5′-GCGTCGACGTCCTGAGGCGGAAAGAA-3′; reverse primer: 5′-CCAGCGCTGGGGTATGTAGGCGGTGCTAC-3′) at 71 °C annealing temperature, for 35 cycles. Subsequently, the 2024-bp target fragment and the vector pNF-κB-SEAP were digested with the restriction enzymes SalI and AfeI (Fermentas) and then joined by T4 DNA ligase (TAKARA) (Fig. 1a). Lastly, the recombinant plasmid, pNF-κB-SEAP-Neo, was sequenced (Beijing Genomics Institute).

### Establishment of RAW264.7 cells stably expressing NF-κB-dependent SEAP reporter gene

RAW264.7 cells obtained from the Cell Bank of the Chinese Academy of Sciences (Shanghai, China),were cultured in RPMI 1640 medium (Hyclone) with 10% heat-inactivated foetal bovine serum (FBS; Hyclone) at 37 °C under 5% CO_2_ and passaged every 2 days. At the third passage, the cells were inoculated into 6-well plates with 1 × 10^6^ per well and cultured overnight until the cells reached 80%–90% confluence. The plasmid pNF-κB-SEAP-Neo was extracted with an Endo free Plasmid Mini preparation Kit (Omega) and its concentration was determined using a Nanodrop (Thermo Fisher) spectrophotometer. The extracted pNF-κB-SEAP-Neo was transfected into RAW264.7 cells using Fugene® HD Transfection Reagent (Promega). To form the transfection complex, the medium was removed from the wells and 2 mL fresh RPMI 1640 medium was added. Following this, 2 μg plasmid and 8 μL Transfection Reagent diluted in 100 μL RPMI 1640 medium without serum were mixed for 1 s and added to the wells in a drop-wise manner, swirled to ensure that the transfection complex covered the entire plate surface, and incubated for 15 min at room temperature.

After transfection with pNF-κB-SEAP-Neo plasmid for 24 h, RAW264.7 cells were selected with 200 μg/mL G418 (Ameresco). Fresh medium containing 200 μg/mL G418 was replenished every other day, for 15 days. The positive clones of RAW264.7-pNF-κB-SEAP cells were cultured and proliferated in RPMI 1640 medium with 10% FBS and 200 μg/mL G418, at 37 °C under 5% CO_2_, and designated as RAW264.7-pNF-κB-SEAP cells.

### Alkaline phosphatase activity assay

Pure CIAP (Takara) was serially diluted in ultrapure-water to obtain a series: 0.8 × 10^-2^, 1.6 × 10^-2^, 2.4 × 10^-2^, 3.2 × 10^-2^, and 4.0 × 10^-2^ U/mL. The substrate reaction buffer was prepared by dissolving PNPP (Sigma-Aldrich) in 1 mg/mL diethanolamine (DEA) buffer (0.1moL/L DEA, 1moL/L MgCl2, pH 9.8). Subsequently, 50 μL substrate was mixed with 50 μL standard alkaline phosphatase solution and left to react at 37 °C for 30 min in the dark. The microplate was then placed in an ice-bath to end the reaction. The absorbance of the product was read at 405 nm (OD_405_) and ultrapure-water is used to adjust zero, using a full-wavelength microplate reader (Thermo). Taking OD_405_ as the abscissa and CIAP activity as the ordinate, a standard curve for SEAP activity was obtained.

When analysing SEAP activity in the culture supernatant, we collected the culture supernatant (150 μL per well) and heated it at 65 °C for 5 min to inactivate other alkaline phosphatases in the cells and the serum, and mixed 50 μL culture supernatant with 50 μL substrate reaction buffer at 37 °C and left it to react for 30 min in the dark. The microplates were then placed in an ice-bath to end the reaction. Absorbance of the product was read at 405 nm (OD_405_) and fresh medium without SEAP was used to adjust zero, using a full-wavelength microplate reader (Thermo). The absorbance was considered directly proportional to the activity of SEAP in the cell supernatant.

### Optimizing the drug screening system with RAW264.7-pNF-κB-SEAP

To determine the optimum number of cells in the initial inoculum for drug screening, the cells were inoculated in a 96-well plate at concentrations of 10,000, 20,000, 40,000, 80,000 and 160,000 cells/well, cultured for 8 h, and incubated in 150 μL/well of high-glucose DMEM without phenol red (Hyclone) and with or without lipopolysaccharide (LPS) (100 ng/mL) for 24 h. SEAP activity in the culture supernatant was measured by microplate spectrometry.

To determine a suitable NF-κB agonist for RAW264.7-pNF-κB-SEAP cells, the cells were treated with NF-κB pathway agonists such as LPS, PolyI:C, and TNF-α at different concentrations. SEAP activity in the culture supernatant was measured at different time points using microplate spectrometry.

The optimal quantity of RAW264.7-pNF-κB-SEAP cell inoculum was added to a 96-well plate and cultured in high-glucose DMEM without phenol red at 37 °C and 5% CO_2_ for 8 h. The cells were then treated for 24 h with 0.1, 1, 10, or 100 ng/mL LPS (Sigma-Aldrich); 1, 10, or 100 μM PolyI:C (Amacia); 1, 10, or 100 ng/mL TNF-α (Peprotech); or in the absence of an agonist as the control. Following this, the concentrations of SEAP in the samples and control were determined and relative expression of SEAP (fold) was calculated using the equation:

$$ \mathrm{The}\kern0.5em \mathrm{relative}\kern0.5em \mathrm{expression}\kern0.5em \mathrm{of}\kern0.5em \mathrm{SEAP}\kern0.5em \left(\mathrm{fold}\right)=\frac{\mathrm{SEAP}\kern0.5em \mathrm{concentration}\kern0.5em \mathrm{in}\kern0.5em \mathrm{the}\kern0.5em \mathrm{sample}}{\mathrm{SEAP}\kern0.5em \mathrm{concentration}\kern0.5em \operatorname{in}\mathrm{the}\kern0.5em \mathrm{control}} $$

Similarly, after inoculation in a 96-well plate and incubated in high-glucose DMEM without phenol red at 37 °C and 5% CO_2_ for 8 h, the cells were treated with 100 ng/mL LPS, 10nM PolyI:C, or 10 ng/mL TNF-α for 6, 12, or 24 h, or without any agonist as the control. SEAP concentration in the samples and control were determined and the relative activity of SEAP (fold) was calculated using the above equation.

To determine a NF-κB inhibitor positive control for the drug screening system, RAW264.7-pNF-κB-SEAP cells were treated with NF-κB inhibitors pyrrolidinedithiocarbamate (PDTC) [19] or (2E)-3-[[4-(1,1-Dimethylethyl) phenyl] sulfonyl]-2-propenenitrile (BAY117085) [20].

After the cells were inoculated in a 96-well plate and cultured with high-glucose DMEM without phenol red at 37 °C and 5% CO_2_ for 2 h, RAW264.7-pNF-κB-SEAP cells with the optimal quantity of inoculum were treated with PDTC (Beyotime) and BAY117085 (Beyotime) at the concentrations of 0.1, 1, 10, and 100 μM for 6 h or with 1‰ DMSO (v/v) for 6 h as the control. Then, they were cultured in high-glucose DMEM without phenol red containing 100 ng/mL LPS for 24 h. SEAP concentrations in the samples and control were determined and the relative expression of SEAP (fold) was calculated using the above equation.

### The screening compounds

Using our established system, we screened several series of compounds, including compounds labelled W (N-(prop-2-ynyl)-4-(substituted phenylcarbonylamino) benzamides) [21], compounds labelled K (2-arylisoquinoline-1, 3(2H, 4H)-diones [22], compounds labelled Z (N-aryl salicylamides) [23], compounds labelled X (diaryl urea) [24], and compounds labelled S (5-(2-aminobenzo[d] hiazole-6-yl)-2-methoxy-3-(phenylsulfonylamino) benzamides) [25].

Our final screened compound W10 was synthesised as follows. To the solution of methyl 4-(chlorocarbonyl) benzoate (0.60 g, 3 mmol) in anhydrous THF (10 ml), a solution of 4-amino-N-(prop-2-yn-1-yl) benzamide (0.20 g, 1 mmol) and diisopropylethylamine (1.00 ml, 0.006 mol) in anhydrous THF (10 ml) was added drop-wise at 0 °C. The reaction mixture was then stirred for 3.5 h at room temperature. After evaporating the solvent, the residue was dissolved in ethyl acetate (20 ml). The mixture was washed with water, followed by washing with 1 M sodium hydroxide, 1 M diluted hydrogen chloride, and brine; dried over sodium sulphate; and filtered. The filtrate was evaporated under reduced pressure to afford a white solid of 0.21 g. A yield of 67.0% was obtained. W10 is methyl 4-((4-(prop-2-ynylcarbamoyl) phenylcarbamoyl) benzoate with its melting point between 240 °C and 242 °C. 1H-NMR (400 MHz, DMSO-d6): δ 10.66 (s, 1H), 8.87 (s, 1H), 8.10 (s, 4H), 7.89 (s, 4H), 4.06 (s, 2H), 3.90 (s, 3H), 3.13 (s, 1H). HRMS m/z Calculated for C19H17N2O4 [M + H] + 337.1188, found 337.1178 [21].

### Screening of 130 compounds to find NF-κB inhibitors and verification of the inhibitory effect of the screened compound (W10)

After RAW264.7-pNF-κB-SEAP cells at the optimal inoculum concentration were added to 96-well plates and cultured in high-glucose DMEM without phenol red at 37 °C and 5% CO_2_ for 2 h, these cells were treated with each of the 130 compounds at the concentration of 10 μM for 6 h or with 1‰ DMSO (v/v) for 6 h as the control. The cells were then cultured in high-glucose DMEM without phenol red, containing 100 ng/mL LPS for 24 h. Lastly, SEAP concentration in the samples and control was calculated and the inhibition ratio of compounds to NF-κB was determined using the following equation:

$$ \mathrm{Inhibitory}\ \mathrm{ratio}\ \left(\%\right) = \left(1-\mathrm{relative}\ \mathrm{activity}\ \mathrm{of}\ \mathrm{SEAP}\right) \times 100 $$

We tested the inhibitory effect of W10 on the transcription of genes promoted by NF-κB in tumour cell lines where NF-κB is known to be continuously activated. HepG 2 and HeLa cell lines obtained from the Cell Bank of the Chinese Academy of Sciences (Shanghai, China) were chosen for this purpose. After inoculation in 6-well plates at a concentration of 3 × 10^5^ cells/well and incubation in high-glucose DMEM at 37 °C with 5% CO_2_ for 12 h, HepG 2 and HeLa cells were each, treated with 10^-5^ M W10, 10^-5^M PDTC or 1‰ DMSO (v/v) for 24 h or 48 h respectively. We extracted total RNA from these samples and tested the transcription of cyclooxygenase (COX)-2, monocyte chemoattractant protein (MCP) 1, and intercellular adhesion molecule (ICAM) 1, which are known to be promoted by NF-κB.

### Scratch assay

We inoculated Hela cells at a concentration of 3 × 10^5^ cells/well in 6-well plate and incubated in high-glucose DMEM at 37 °C with 5% CO_2_ for 12 h, marking off in the cells with 10 μL tip as straight as possible. The Hela cells with mark-off line were treated with 1‰ DMSO (v/v) and 10^-5^ M W10 in high-glucose DMEM without FBS for 24 h or 48 h respectively. The scratches in cells were observed and photographed with inverted microscope (Nikon Eclipse Ri) at 24 h and 48 h. The 3 fixture positions were selected then photographed and measured width of the scratches at the positions at 24 h and 48 h, statistical the variation of scratches width.

### Real-time quantitative polymerase chain reaction

Total RNA from the samples was extracted with TriPure Isolation Reagent (Roche) as follows: After removing the medium, 1 mL reagent was added into each well, incubated for 5 min, and then transferred to a fresh 1.5-mL Eppendorf tube, where chloroform (0.2 ml per 1 ml of TriPure) was added. Then, the tubes were vortexed for mixing, followed by centrifugation at 12,000 g and 4 °C for 15 min till the liquid separated into 3 phases. The aqueous phase was transferred carefully to a new tube, mixed with an equal volume of isopropanol, vibrated gently, and incubated at -20 °C overnight to precipitate RNA. The mixture was centrifuged at 12,000 g and 4 °C for 15 min; then, isopropanol was discarded and 75% ethanol was added. The mixture was subjected to gentle vibration and centrifuged again at 12,000 g and 4 °C for 15 min. The ethanol was then removed and placed at room temperature until the RNA was dry and then retrieved with 10 μL diethylpyrocarbonate in water. The quantity of the RNA was estimated using a Nanodrop (Thermo Fisher) spectrophotometer.

Using RNA from the samples as template, cDNA was prepared and amplified (First Strand cDNA Synthesis Kit, Thermo). The amplification system included 5 μg total RNA and 1 μL oligo (dT) primer, and the total volume made up to 12 μL with water. This system was incubated at 65 °C for 5 min, cooled quickly on ice and the following reagents were added to a final volume of 20 μL: 4 μL 5× Reaction Buffer, 1 μL RiboLock RNase Inhibitor (20U/μL), 2 μL dNTP Mix (10 mM), and 1 μL RevertAid M-MuLV RT (200U/μL). The amplification mixture was mixed gently, centrifuged instantaneously, and incubated at 42 °C for 60 min. The whole amplification reaction was terminated at 70 °C for 5 min.

In HeLa and HepG 2 cell lines, the transcription of genes encoding MCP1, COX-2, and ICAM1 was detected using real-time quantitative polymerase chain reaction (RT- qPCR), with iQ5 (Bio-Rad, Hercules, USA) and FastStart Universal SYBR Green Master (ROX) (Roche) for 40 cycles. The relative gene expression normalized by β-actin was calculated by the 2-ΔΔCT method. Details of the primers used are shown in Additional file 1: Table S1.

### Statistics

All experiments were repeated 3 times, with 3 replicates per analysis. Significance of difference between samples and control was determined using the 1 way-ANOVA. All raw values were transformed with EXCEL 2010 and statistical analysis was performed using Prism 6.0 software.

## Results

### Establishment of RAW264.7-pNF-κB-SEAP cells stably expressing NF-κB-dependent SEAP reporter gene

The reporter gene vector (Clontech) is a transient-transfection plasmid and does not contain the eukaryotic resistant gene for stable transfection. Therefore, it is not suitable for establishment of the drug screening system (Fig. 1a). There are 2 restriction sites (SalI and AfeI) downstream of the SEAP transcript in the vector pNF-κB-SEAP. We amplified the fragment of neomycin resistance geneNeoR/KanR ORF and TK-PA-terminator in the vector pEGFP-N1,inserted the fragment into downstream of SEAP transcript of pNF-κB-SEAP (Fig. 1a). The amplified fragment was 2,042 bp long (Fig. 1b). Both pNF-κB-SEAP and the amplified fragment were digested with SalI and AfeI (Fig. 1b) and then linked to construct the recombinant SEAP reporter gene vector (pNF-κB-SEAP-Neo) (Fig. 1a and b). The positive recombinant including pNF-κB-SEAP-Neo was selected on Luria Bertani agar with ampicillin and then proliferated in order to extract endotoxin-free plasmid for eukaryotic transfection. DNA sequencing showed that there was no mutation in the amplified fragment of the recombinant vector pNF-κB-SEAP-Neo.

The extracted pNF-κB-SEAP-Neo was transfected into RAW264.7 cells. By selection with fresh medium containing 200 μg/mL G418for 15 days,the RAW264.7 cells stably expressing the SEAP reporter gene (RAW264.7-pNF-κB-SEAP cells) were established.

### Alkaline phosphatase activity assay

**Fig. 2:**
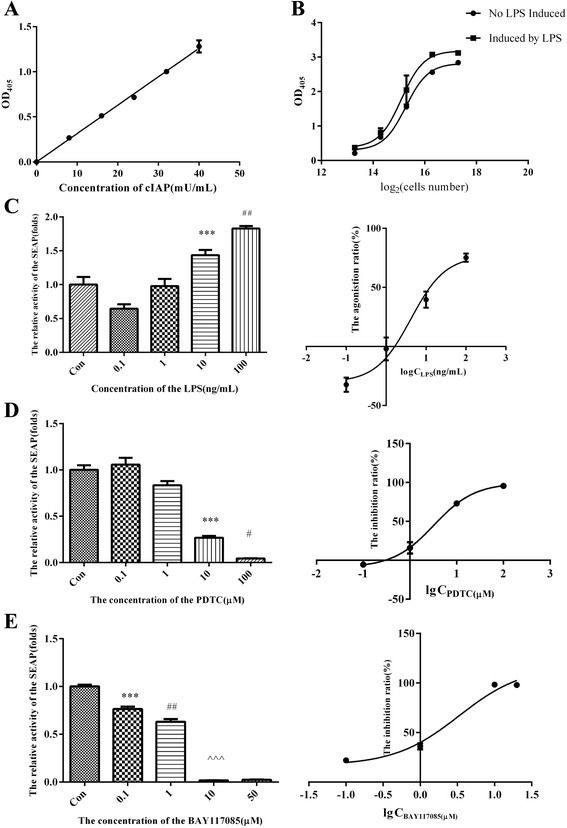
Identification of standard screening method based on RAW264.7 Cell line stably expressing NF-κB-dependent SEAP reporter Gene. **a** Standard curve of alkaline phosphatase, y = 0.0315x, R^2^ = 0.9947. **b** Relationship between OD_405_ and cell number/well of Raw264.7 cells stably expressing NF-κB dependent SEAP reporter gene (RAW264.7-pNF-κB-SEAP cells)treated with or without LPS. R^2^ = 0.9395 (the cells were treated with LPS), and R^2^ = 0.9910 (the cells were treated without LPS). **c** The effect of RAW264.7-pNF-κB-SEAP cells were treated with different concentrations of LPS for 24 h menifested as column chart of relative activity of SEAP and dose-response stimulation curve with common logarithm of LPS concentration as the ordinate and the stimulation ratio (%) as the abscissa, R^2^ = 0.9643. **d** The RAW264.7-pNF-κB-SEAP cells were treated with PDTC at concentrations of 0.1, 1, 10, and 100 μM or 1‰ DMSO (v/v) (as control) for 6 h and then treated with LPS in 100 ng/mL for 24 h. The effect of PDTC on RAW264.7-pNF-κB-SEAP cells was manifested as column chart of relative activity of SEAP and the dose-response inhibition curve between the log_10_ concentrations of PDTC and the inhibition ratio calculated from SEAP relative activity, R^2^ = 0.9802, their relation according to the common logarithmic equation. **e** The RAW264.7-pNF-κB-SEAP cells were treated with BAY117085 at concentrations of 0.1, 1, 10, and 50 μM or 1‰ DMSO (v/v) (as control) for 6 h and then treated with LPS in 100 ng/mL for 24 h. The effect of BAY117085 on RAW264.7-pNF-κB-SEAP cells was manifested as column chart of relative activity of SEAP and the dose-response inhibition curve between the log_10_ concentrations of BAY117085 and the inhibition ratio calculated from SEAP relative activity, R^2^ = 0.9735, their relation according to the common logarithmic equation. The “*” indicates that the column had significant difference compared with the control, the others marked as “#” and “^” indicate that the column showed significant difference compared with the column on the left

SEAP reporter gene expression is indicated by its activity; therefore, the concentration of SEAP was determined as its activity per unit volume. Since there is a high level of similarity between SEAP and calf intestine alkaline phosphatase (CIAP), the standard curve of SEAP activity was prepared using CIAP. In the standard curve, the abscissa denotes different concentrations of the standard substance, CIAP; the ordinate denotes OD_405_, indicating the activity of alkaline phosphatase under different concentrations of CIAP (Fig. 2a and Additional file 2). Since CIAP is highly similar to SEAP except for the signal peptide, the ordinate denotes OD_405_, it also reflects the activity of alkaline phosphatase under different concentrations of SEAP. The equation of standard curve is y = 0.000315x and R^2^ = 0.9947. Therefore, SEAP activity in the culture supernatant can be calculated using this standard curve or equation using their OD_405_ values.

### Construction of a drug screening system based on the recombined cell line, RAW264.7-pNF-κB-SEAP

In order to select the optimal initial inoculum of cells for anti-inflammatory drug screen, the activity of SEAP in culture supernatant of the RAW264.7-pNF-κB-SEAP cells with different inocula per cell was determined in the presence or absence of LPS (100 ng/mL) for 24 h. The results showed that the relationship between the log_2_ of initial inoculum and OD_405_ was consistent with the agonist dose-response curve, when the initial inoculum ranged from 20,000 to 160,000cells/well (Fig. 2b and Additional file 2). Furthermore, when the initial inoculum was 40,000cells/well, the OD_405_ in the presence of LPS was significantly higher than that in the absence of LPS. Therefore, we selected 40,000 cells of RAW264.7-pNF-κB-SEAP cells/well in a 96-well plate for subsequent experiments. The relationship between log_2_ of initial inoculum and OD_405_ was consistent with the agonist-response curve as well, which further indicated that the RAW264.7-pNF-κB-SEAP cells were successfully established, stable, and can be applied to the screening of NF-κB-dependent anti-inflammatory compounds.

To activate NF-κB in this cell model, we treated the RAW264.7-pNF-κB-SEAP cells with 3 NF-κB pathway agonists: LPS, TNF-α, and polyI:C for 24 h. The results showed that there was no difference after TNF-α treatment (Additional file 1: Figure S1A and Additional file 2), while SEAP secretion was significantly up-regulated after treatment with 10nM polyI:C (Additional file 1: Figure S1B and Additional file 2) and 10 and 100 ng/mL LPS (Fig. 2c and Additional file 2), compared with the control (DMSO group). We found that the relation between excitation ratio of SEAP expression and the concentration of LPS was according to the excitation-response curve, R^2^ = 0.9643, and this result indicated that the effect of LPS on SEAP secretion of RAW264.7-pNF-κB-SEAP cells was dose-dependent (Fig. 2c and Additional file 2). However, as traditional agonists to Toll-like receptor -3 and TNFR, activation of polyI:C and TNF-α to NF-κB should be present in this reporter gene system; therefore, we tested for the effects of LPS, polyI:C, and TNF-α on SEAP at different time points. The results showed that SEAP secretion was up-regulated only after treatment with 10nM polyI:C and 10 ng/mL TNF-α for 12 h, while there was significant up-regulation at 3 time points after treatment with 100 ng/mL LPS, compared with their corresponding controls (DMSO group) (Additional file 1: Figure S1C) and Additional file 2. Therefore, the optimal agonist of NF-κB was LPS, at 100 ng/mL with treatment duration of 24 h, respectively (Fig. 2c and Additional file 2).

In order to select a NF-κB inhibitor as positive control in the drug screening system for anti-inflammatory compounds, the RAW264.7-pNF-κB-SEAP cells were pre-treated with NF-κB inhibitors, PDTC and BAY117085 for 6 h before treatment with 100 ng/mL LPS for 24 h. The results showed that PDTC significantly down-regulated SEAP secretion by these cells compared to the control group, at concentrations ranging from 10 to 100 μM (Fig. 2d and Additional file 2), while BAY117085 significantly decreased SEAP secretion by these cells compared to the control group at concentrations ranging from 0.1 to 50 μM (Fig. 2e and Additional file 2). Although the inhibitory effect of BAY117085 seemed to be superior to that of PDTC, some dead cells were observed with BAY117085 at concentration of 100 μM. Therefore, PDTC was selected as the positive control in the drug screening system for anti-inflammatory compounds. In addition, as the inhibition ratio of 10 μM PDTC was nearer to 50%, the concentration of 10 μM PDTC was used in subsequent screening.

Thus, the drug screening system on the basis of the RAW264.7-pNF-κB-SEAP was established. The procedure of screening was as follows: the RAW264.7-pNF-κB-SEAP cells were inoculated in the 96-well plate at a concentration of 40,000cells/well and incubated with high-glucose DMEM without phenol red at 37 °C for 2 h to allow adherence. These cells were then treated with 100 ng/mL LPS for 24 h after pre-treatment with 10 μM test compounds, 1‰ DMSO (blank control), and 10 μM PDTC (positive control) for 6 h. The activity of SEAP in the culture supernatant was determined. Lastly, the inhibitory ratio of compounds was calculated as compared to blank control.

### Screening of 130 compounds as NF-κB inhibitors using the drug screening system and verification of the inhibitory effect of the screened compound W10

Using our new drug screen system, we screened 130 compounds. The results showed that 13 compounds had remarkable inhibitory effect on the secretion of SEAP (Table 1 and Additional file 2). Of these, 8 compounds (*P* < 0.01) (Table 1 and Additional file 2) were analysed once again. The results showed that there was no difference in their inhibition ratio between the primary and the repeat screens (Table 2 and Additional file 2). The inhibition ratio of the compound W10 was stable in the 2 screenings (Table 2 and Additional file 2). In contrast, the inhibition ratio of the compound S15 was significantly increased in the repeat than in the primary screen (Table 2 and Additional file 2). We chose compound W10 to carry out subsequent studies.

**Table 1:** The inhibition ratio of the 130 chemicals

Compound No.	Inhibition ratio (%)	Compound No.	Inhibition ratio (%)	Compound No.	Inhibition ratio (%)
w1	-11.65 ± 1.29	k26	-14.74 ± 2.00	z4	-44.29 ± 19.53
w2	-23.07 ± 8.68	k27	-16.02 ± 1.17	z5	-39.52 ± 17.50
w3	-9.38 ± 1.53	k28	1.64 ± 3.12	z6	-19.94 ± 3.85
w4	-2.92 ± 2.03	k30	-14.45 ± 3.33	z7	-35.50 ± 16.85
w5	-12.74 ± 2.48	k32	-13.99 ± 2.33	z8	-29.85 ± 15.10
w6	-6.40 ± 2.60	k34	6.06 ± 2.06	z9	-35.72 ± 16.32
w7	2.21 ± 3.22	k35	-9.43 ± 2.00	z10	-13.67 ± 11.87
w8	4.85 ± 5.55	k36	4.81 ± 8.98	z11	-21.48 ± 7.93
w9	6.41 ± 7.79	k37	-4.56 ± 0.75	z12	-22.78 ± 7.29
W10	27.20 ± 5.91^***^	k38	-6.22 ± 1.40	z13	-38.60 ± 16.45
w11	12.99 ± 0.34^**^	k33	-4.24 ± 3.08	z14	-42.24 ± 19.10
w12	4.49 ± 8.32	k40	-4.84 ± 0.62	z15	-9.32 ± 10.93
w13	-2.24 ± 2.98	x1	-10.09 ± 0.37	z16	-17.9 ± 13.51
w14	-2.85 ± 2.72	*x*2	-13.39 ± 0.83	z17	-16.95 ± 15.87
w15	42.95 ± 28.87^*^	x3	-6.83 ± 2.87	z18	-10.58 ± 10.06
w19	15.76 ± 5.56^**^	x4	-9.21 ± 3.05	z19	6.12 ± 6.76
w20	29.82 ± 10.13^**^	x5	-2.57 ± 1.20	z20	-13.51 ± 4.73
w21	-10.04 ± 1.59	x6	-8.41 ± 0.48	z21	-4.97 ± 4.55
w22	-12.64 ± 4.55	x8	-3.20 ± 0.43	z22	-2.92 ± 4.35
w23	-12.17 ± 3.76	x9	9.43 ± 1.85^*^	s1	15.23 ± 1.85^**^
k1	-8.45 ± 3.57	x10	-4.75 ± 0.60	s2	6.82 ± 1.28
k2	-2.86 ± 1.86	x11	-3.74 ± 0.85	s3	26.57 ± 6.43^***^
k3	-5.10 ± 1.92	x12	-3.49 ± 0.63	s4	10.68 ± 3.97^*^
k4	-11.37 ± 3.07	x13	-6.20 ± 9.91	s5	-0.58 ± 3.33
k5	-4.99 ± 1.116	x14	-14.30 ± 9.40	s6	-3.90 ± 6.65
k6	-2.10 ± 1.52	x15	-20.12 ± 16.57	s7	-11.21 ± 2.75
k7	-4.36 ± 2.82	x16	-37.86 ± 6.55	s8	-14.82 ± 6.92
k8	-2.22 ± 3.39	x17	-31.71 ± 2.97	s9	-9.54 ± 3.42
k9	-6.56 ± 2.32	x18	-41.94 ± 8.21	s10	-23.73 ± 7.42
k10	-4.30 ± 2.47	x19	-26.51 ± 20.62	s11	-14.65 ± 5.85
k11	-3.05 ± 2.17	x20	-14.09 ± 8.09	s12	-6.06 ± 25.05
k12	5.12 ± 0.42	x21	-25.66 ± 11.07	s13	-11.09 ± 2.86
k13	8.26 ± 2.57	x22	5.25 ± 18.74	s14	-4.31 ± 2.64
k14	9.31 ± 2.81	x23	4.80 ± 1.82	s15	20.42 ± 2.62^***^
k15	5.30 ± 1.41	x24	7.56 ± 9.16	s16	4.57 ± 5.97
k16	12.63 ± 2.95^*^	x25	-7.50 ± 5.60	s17	11.34 ± 1.90
k17	6.19 ± 0.75	x26	3.62 ± 5.16	s18	16.33 ± 9.43^*^
k18	-6.14 ± 0.96	x27	0.19 ± 3.65	s19	-5.27 ± 3.55
k20	3.80 ± 0.79	x28	11.67 ± 10.08	s20	19.63 ± 2.34^***^
k21	-2.91 ± 0.58	x29	-1.12 ± 8.86	s22	6.79 ± 4.18
k22	-0.02 ± 2.30	x30	-3.87 ± 3.32	s23	-6.96 ± 4.91
k23	1.35 ± 1.78	z1	-23.43 ± 13.80	s24	-16.02 ± 6.30
k24	-2.47 ± 2.41	z2	-28.88 ± 17.48	PDTC	50.35 ± 1.36
k25	-4.87 ± 1.48	z3	-19.87 ± 11.88		

The result showed as the format of “Mean ± SEM”

The “*” means that the column had significant difference compared with the control, *P* < 0.05; **P < 0.01; and ***P < 0.001﻿

**Table 2:** Comparison of inhibition ratio of 8 chemicals between the primary screening and the secondary screening

Compound No	Inhibition ratio(%) from 1^st^creening	Inhibition ratio(%) from 2^nd^ screening
w10	27.20 ± 5.91	26.64 ± 1.41
w11	12.99 ± 0.34	23.96 ± 6.22
w19	15.76 ± 5.57	12.44 ± 1.08
w20	29.82 ± 10.13	7.73 ± 1.18
s1	15.23 ± 1.85	10.12 ± 1.24
s3	26.57 ± 6.43	20.35 ± 0.86
s15	20.42 ± 2.62	41.90 ± 0.55
s20	19.63 ± 2.34	22.52 ± 0.81
PDTC	40.97 ± 1.32	52.07 ± 3.45

The results showed as the format of”Mean ± SEM”

**Fig. 3:**
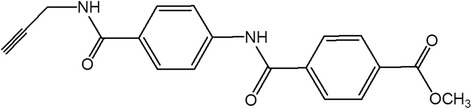
The structure of w10. Methyl 4-((4-(prop-2-ynylcarbamoyl) phenylcarbamoyl) benzoate (w10)

**Fig. 4:**
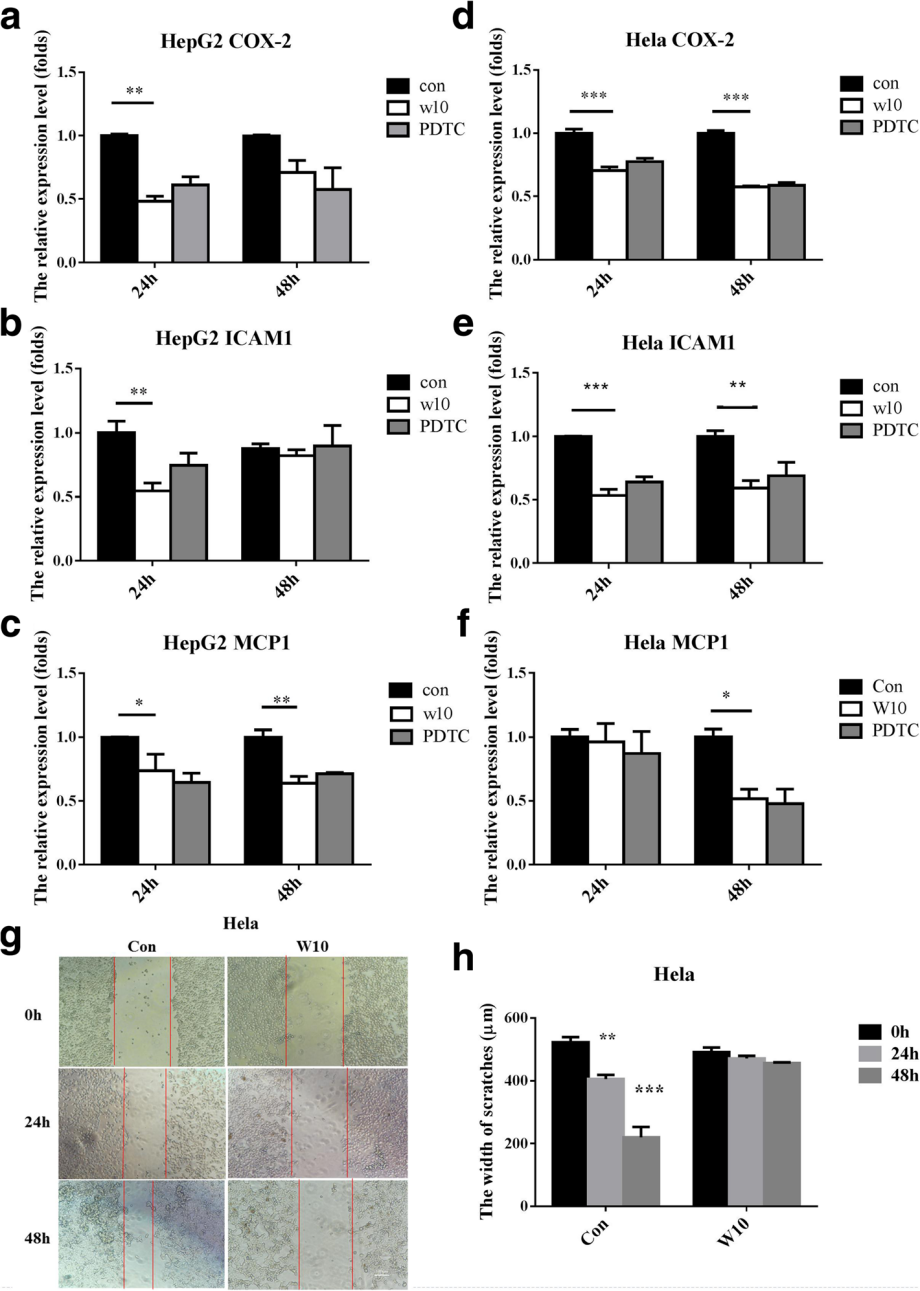
The effectsto HepG2 and HeLa cellstreated with W10 in relative gene and scratches. The relative expression level of COX-2 (**a**), ICAM1 (**b**), and MCP1 (**c**) in HepG 2 cells after treatment with control, PDTC, or W10 for 24 or 48 h. The relative expression level of COX-2 (**d**), ICAM1 (**e**), and MCP1 (**f**) in HeLa cells after treatment with control, PDTC, or W10 for 24 or 48 h. The width of scratchesin Hela cells after treatment with control and W10 for 24 h or 48 h expressed in photograph (**g**) and histogram (**h**). The data are expressed as mean ± SEM from 3 independent experiments. The “*” indicates that the column had significant difference compared with the control in group

The compound structure of W10 is shown in Fig. 3. We planned for further studies on W10 at the molecular mechanism of the compound stable inhibition on the NF-κB. RAW264.7 cells were treated with W10 for 2 h and then with LPS for 6, 12, and 24 h. Transcription of COX-2 and IL6 was activated by NF-κB. The result showed that the transcription of COX-2 and IL6 was up-regulated by LPS, but W10 did not inhibit their up-regulation induced by LPS (Additional file 1: Figure S2 and Additional file 2). The transcription of COX-2, ICAM1 and MCP1 was significantly inhibited in HepG 2 cells after treatment with W10 for 24 h, whereas, transcription inhibition by MCP1 remained for only 48 h (Fig. 4a, b, and c and Additional file 2). The transcription of COX-2 and ICAM1 was significantly inhibited in HeLa cells after treatment with W10 for 24 h and this inhibition remained for 48 h. Additionally, the transcription of MCP1 was significantly inhibited in HeLa cells after treatment with W10 for 48 h (Fig. 4d, e, and f and Additional file 2). As the transcription of ICAM1 and MCP1 were inhibited with W10, we detected whether W10 inhibited the migration of tumour cells with scratch testing. The results showed that the scratches in HeLa cells were significant narrow down in control group for 24 h and 48 h. However, the scratches in HeLa cells have not vary significantly for 24 h and 48 h in W10 treated group (Fig. 4g and h and Additional file 2).

## Discussion

The most important parameter for a drug screening system is stability. To this end, we constructed a stable recombinant cell line, selected a reporter gene with a prolonged half-life, used standard detection methods for the reporter gene, and selected an optimal agonist and inhibitor for this system. Although a drug screening system based on instantaneous transfection of reporter gene has been previously reported, we constructed a stable reporter gene recombinant cell line, since transfection of the RAW264.7 cell line selected for this system is a challenge. We constructed a stable reporter gene recombinant cell line to provide a foundation for stability of the screening system. Similarly, while selecting the reporter gene, we chose SEAP rather than luciferase, which is otherwise popular in drug screening methods. Due to its sensitivity and ease of detection, luciferase is suited to testing cells in which a transcription promoter is momentarily activated or inhibited [26]. However, signal pathway activation and cascade mechanisms vary. For example, persistent effect as seen with LPS activating NF-κB through binding with TLR4, pulsating effect as seen with TNF-α activating NF-κB through binding with TNFR [27], feedback effect as seen with HDL inhibiting proinflammatory gene transcription by activating ATF3 [28]. These effects occur in different periods and are difficult to detect in a single test based on luciferase. Other than the instant effect, we focused on the complete effect of the compound on cells within a period, therefore we chose the reporter gene SEAP which has a longer half-life and lower distinguishing ability, to construct the drug screen system [29]. For SEAP testing, we established a standard curve of alkaline phosphatase activity that improves the stability of the drug screening system. The standard curve of alkaline phosphatase activity translates the screening results into absolute, quantified values and provides uniformity to the screening results from different time points. In the screening process, we chose an optimal agonist and inhibitor of NF-κB to treat the reporter gene recombinant cell line. LPS was found to significantly induce NF-κB activation for a long period (24 h). The significant up-regulation of SEAP expression was sustained throughout this period. This persistent and significant activation of NF-κB authenticates the inhibitory effect of the candidate compounds and diminishes false-negative error. PDTC is recognized as a typical NF-κB inhibitor. This was chosen as the optimal inhibitor of NF-κB in our drug screening system. During screening, we treated cells with PDTC at the same concentration as candidate compounds, which was near to the IC_50_ of PDTC for NF-κB. While the samples treated with PDTC were taken as positive control, we could gauge whether inhibition of SEAP expression by PDTC skewing the results indicated if the cells are unusually sensitive due to age, mutation or other reasons. PDTC as positive control compound helped ensure that the drug screening system was stable. Besides, we tested the inhibition of NF-κB by the compound W10 with RT-qPCR, which showed that W10 inhibited transcription of genes regulated by NF-κB and the inhibition of W10 remained for 48 h. Testing and verification of transcription of genes promoted by NF-κB is working for maintaining stability of the drug screening system. After screening 130 compounds using the system, we evaluated stability of the system in terms of ease to operate and perform the test, while maintaining control.

From our study, we found that W10 did not inhibit the transcription of genes downstream of NF-κB activated by LPS; rather, W10 inhibited the transcription of genes downstream of the NF-κB signal in tumour cell lines that NF-κB persistently activated, and part of the inhibitory effect persisted until 48 h. These results indicated that W10 is specific to NF-κB. LPS induces gene transcription not only via NF-κB; W10 specifically inhibited the up-regulation of genes from NF-κB. Therefore, W10 did not inhibit the up-regulation of genes induced by the LPS signal on the whole. This phenomenon shows that the drug screening system we constructed is highly specific to its target and therefore, W10 can be allied to cancer research and therapy.

## Conclusion

We constructed a drug screening system targeting the transcription factor NF-κB, based on the cell line RAW264.7. In this drug screening system, unstable factors were eliminated by standardizing each index and choosing the optimal control, which increased the stability and accuracy of the drug screening system, allowing for a simple and fast assay. The compound W10 were selected with the drug screening system we constructed. In our further studies, W10 was detected to inhibit the transcription of COX-2, ICAM1 and MCP1 in Hela and HepG2 and inhibit the migration of Hela cells.

## Additional files

Additional file 1: Table S1, Figure S1 and Figure S2
Additional file 2: Supporting data for Fig. 2, Fig. 4, Table 1, Table 2, Additional file 1: Figure S1 and Figure S2. (XLSX 66 kb)

## Abbreviations

BAY11-7085: (2E)-3-[[4-(1,1-Dimethylethyl)phenyl]sulfonyl]-2-propenenitrile
CIAP: Calf intestine alkaline phosphatase
COX-2: Cyclooxygenase-2
DEA: Diethanolamine
ICAM1: Intercellular adhesion molecule
IL-1β: Interleukin-1β
LPS: Lipopolysaccharide
MCP1: Monocyte chemoattractant protein 1
NF-κB: Nuclear factor-κB
PDTC: Pyrrolidinedithiocarbamate
PNPP: P-nitrophenyl phosphate
SEAP: SEAP
TNF-α: Tumour necrosis factor TNF-α
