# Supplementary material for: Identification and application of anti-inflammatory compounds screening system based on RAW264.7 cells stably expressing NF-κB-dependent SEAP reporter gene
Source: BMC Pharmacol Toxicol. 2017 Jan 18;18:5. doi: 10.1186/s40360-016-0113-6 (PMC5242024; doi:10.1186/s40360-016-0113-6)
Supplement: Additional file 1: — Table S1, Figure S1 and Figure S2 [file 40360_2016_113_MOESM1_ESM.docx]

**Supplement information**

**Table S1. Primer Sequences and optimal qPCR condition of related genes**

|  | **Gene** | **NCBI accession**  **number** | **Primer sequences** |
| --- | --- | --- | --- |
| Human | COX-2 | M90100 | 5’-CCCTTGGGTGTCAAAGGTAA-3’ |
|  |  |  | 5’-GCCCTCGCTTATGATCTGTC-3’ |
|  | ICAM1 | NM_000201 | 5’-GGCCTCAGTCAGTGTGA-3’ |
|  |  |  | 5’-AACCCCATTCAGCGTCA-3’ |
|  | MCP1 | BC009716 | 5’-CATTGTGGCCAAGGAGATCTG-3’ |
|  |  |  | 5’-CTTCGGAGTTTGGGTTTGCTT-3’ |
|  | ACTB | NM_001101 | 5’-CATCTCTTGCTCGAAGTCCA-3’ |
|  |  |  | 5’-ATCATGTTTGAGACCTTCAACA-3’ |
| Mouse | IL-6 | NM_031168 | 5’-CTGCAAGAGACTTCCATCCAG-3’ |
|  |  |  | 5’-AGTGGTATAGACAGGTCTGTTGG-3’ |
|  | COX-2 | NM_011198 | 5’-TTCAACACACTCTATCACTGGC-3’ |
|  |  |  | 5’-AGAAGCGTTTGCGGTACTCAT-3’ |
|  | ACTB | NM_007393 | 5’-GGCTGTATTCCCCTCCATCG-3’ |
|  |  |  | 5’-CCAGTTGGTAACAATGCCATGT-3’ |


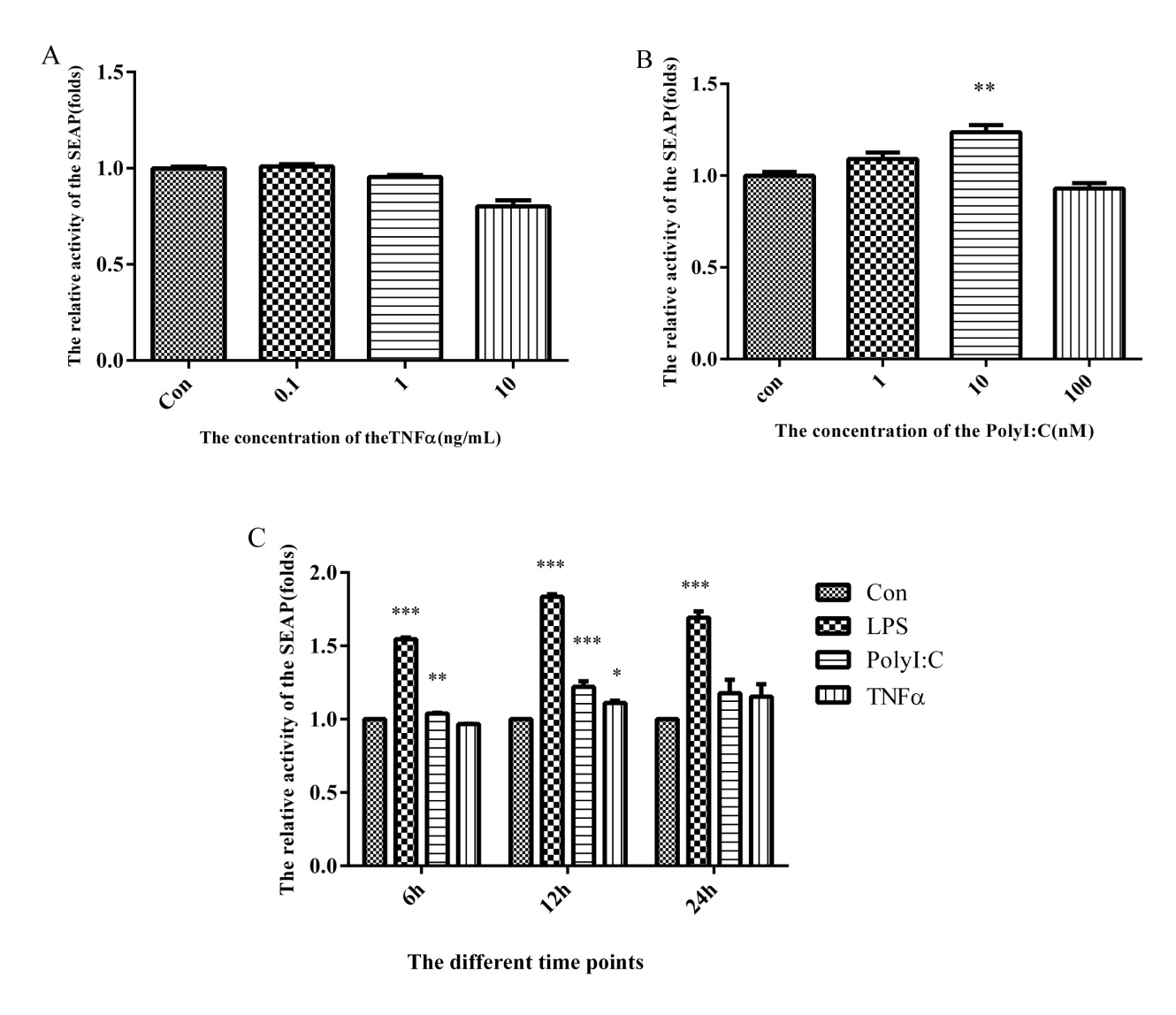


**Figure.S1 The effect of RAW264.7-pNFκB-SEAP cells treated with TNF-α** **and PolyI:C in different concentration and with TNF-α, PolyI:C and LPS on different time point.**

The effect of RAW264.7-pNFκB-SEAP cells were treated with different concentrations of (A) TNF-α, (B) PolyI:C for 24 h menifested as column chart of relative activity of SEAP. The RAW264.7-pNFκB-SEAP cells were treated with 10 ng/mL TNF-α, 10 nM PolyI:C, and 100 ng/mL LPS for 6, 12, and 24 h each. * ,**, and *** indicate that the column had significant differences, P < 0.05, P < 0.01 and P < 0.001.


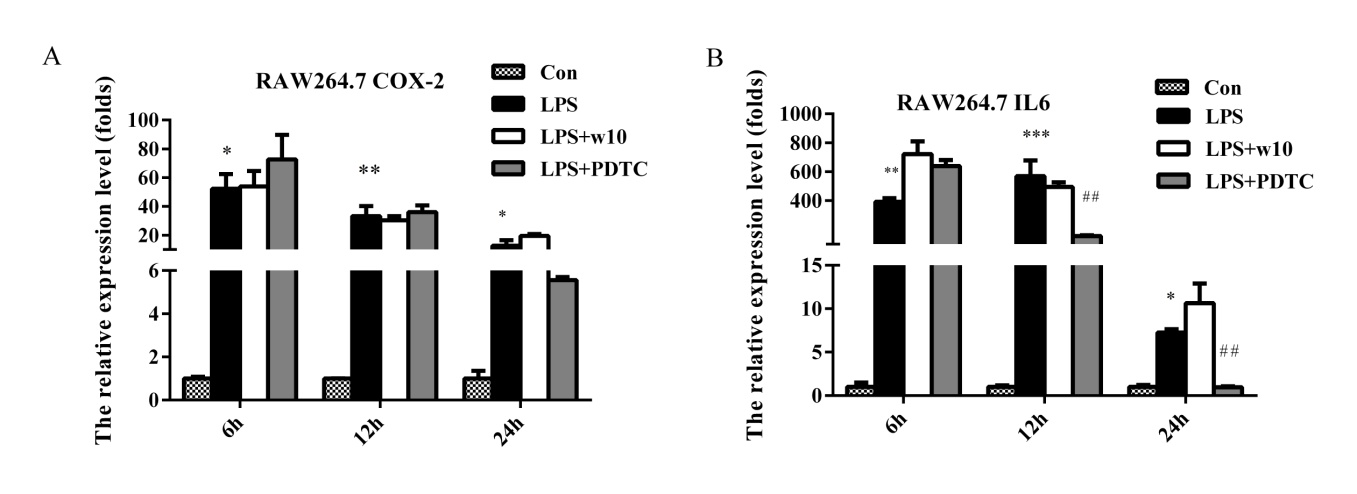


**Figure. S2 The expression of NF-κB downstream genes in Raw264.7 cells treatment with LPS and PDTC or the W10.**

(A) and (B) the expression of COX-2 and IL6 in Raw264.7 cells after treatment with 10 μM PDTC and novel chemical W10 for 6,12, or 24 h in the presence of LPS. The data are expressed as mean±SEM from 3 independent experiments, where the mean value from each was obtained from triplet samples. “*” indicates that the column showed significant difference compared with the control. “#”indicates that the column showed significant difference compared with the column “LPS”.
